# Advances in PARP Inhibitors for Prostate Cancer

**DOI:** 10.3390/cancers15061849

**Published:** 2023-03-20

**Authors:** Steven Tisseverasinghe, Boris Bahoric, Maurice Anidjar, Stephan Probst, Tamim Niazi

**Affiliations:** 1 Department of Radiation Oncology, McGill University, Gatineau, QC J8V 3R2, Canada; 2 Department of Radiation Oncology, McGill University, Montreal, QC H3A 0G4, Canada; 3 Department of Urology, McGill University, Montreal, QC H3A 0G4, Canada; 4 Department of Nuclear Medicine, McGill University, Montreal, QC H3A 0G4, Canada

**Keywords:** prostate cancer, PARP inhibitors, targeted therapy

## Abstract

**Simple Summary:**

Recent practice-changing trials have highlighted the importance of polyadenosine diphosphate-ribose polymerase inhibitors (PARPi) in metastatic castrate resistant prostate cancer (mCRPC). PARP plays a quintessential role in repairing deoxyribonucleic acid (DNA) single-strand breaks by signaling and recruiting the necessary repair machinery to damaged areas. In mCRPC, where mutations are more likely to impair important repair pathways such as the homologous recombinational repair (HRR), remaining PARP repair pathways become critical to cell survival and can be exploited with targeted therapies. The purpose of our review was to compare and contrast recent pivotal trials in this setting and explore future avenues of research.

**Abstract:**

Poly-adenosine diphosphate-ribose polymerase plays an essential role in cell function by regulating apoptosis, genomic stability and DNA repair. PARPi is a promising drug class that has gained significant traction in the last decade with good outcomes in different cancers. Several trials have sought to test its effectiveness in metastatic castration resistant prostate cancer (mCRPC). We conducted a comprehensive literature review to evaluate the current role of PARPi in this setting. To this effect, we conducted queries in the PubMed, Embase and Cochrane databases. We reviewed and compared all major contemporary publications on the topic. In particular, recent phase II and III studies have also demonstrated the benefits of olaparib, rucaparib, niraparib, talazoparib in CRPC. Drug effectiveness has been assessed through radiological progression or overall response. Given the notion of synthetic lethality and potential synergy with other oncological therapies, several trials are looking to integrate PARPi in combined therapies. There remains ongoing controversy on the need for genetic screening prior to treatment initiation as well as the optimal patient population, which would benefit most from PARPi. PARPi is an important asset in the oncological arsenal for mCRPC. New combinations with PARPi may improve outcomes in earlier phases of prostate cancer.

## 1. Introduction

Prostate cancer is the second most common malignancy diagnosed among men globally [1]. Although often curable early on, end-stage disease is often characterized by metastatic castrate resistant prostate cancer (mCRPC). Over the last decade, an improved understanding of underlying disease mechanics has led to tangible improvements in both patient survival and quality of life. Despite these advances, prognosis for end-stage disease remains somber.

Several innovative therapies have been developed for mCRPC over the last decade. Treatment options include next-generation anti-hormonal agents, radiopharmaceuticals, chemotherapy and immunotherapy [2,3,4,5,6,7]. These novel agents have aided patients afflicted with mCRPC through an improved PSA response and a modest survival benefit.

PARPi are a targeted therapy, which aim to exploit differential DNA repair in malignant tumors [8]. Cancer cells, like normal cells, can suffer DNA damage both from endogenous sources such as reactive oxygen species or therapeutic agents such as ionizing radiation or chemotherapy [9]. With severe or cumulative damage, a lethal event inducing cell death can occur [10]. Among the different classes of DNA damage, double-strand breaks (DSB) are arguably the most clinically significant. DSBs can induce chromosomal translocations if misrepaired and cell death if unrepaired. These lesions can arise indirectly from two closely located single-strand breaks (SSB), or during replication fork collapse ensuing from the failed repair of SSB or base damage [11]. Malignant cells also rely on DNA repair mechanisms to ensure their integrity. The two main DNA DSB repair pathways are non-homologous end-joining repair (NHEJ) and homologous recombination repair (HRR) [12]. NHEJ, through Ku enzymes, mediates the capture of both ends of the broken DNA molecules. Both DNA ends are approximated with the help of DNA-PKcs (DNA-dependent protein kinase, catalytic subunit) through the formation of a molecular bridge and are then re-ligated [13]. Meanwhile, HRR promotes high fidelity repair through the unwinding of the damaged DNA helix, invasion of the damaged strands into a homologous DNA duplex molecule and replication using the homologous strand as a template [9]. *BRCA1* and *BRCA2* are tumor suppressor genes that are required for HRR. Mutations in these genes hence can effectively block HRR and subsequently promote genetic instability [14]. PARP-1 have been determined to be hyperactivated in HRR mutated cells and to have a role in reactivating stalled replication forks [15]. Hence, their role is augmented in malignant cells that are beset by DNA damage repair (DDR) gene mutations and consequently have limited DNA repair capacity. Consequently, PARP inhibition in the setting of homologous recombination deficiency (HRD) can lead to synthetic lethality, where their combined deleterious effects on DNA repair culminate in cell death [14]. Interest in exploiting synthetic lethality in mCRPC has further increased, with higher incidental findings of germline mutations in DNA damage repair (DDR) genes in men with mCRPC, currently estimated between 11–33% [16].

In this paper, we seek to discuss in depth the function, utility and efficacy of PARP inhibition in mCRCP. We intend to summarize recent relevant studies on this topic, as well as new and exciting upcoming trials and the overall potential for the use of this class of agent.

## 2. Materials and Methods

**Figure 1:**
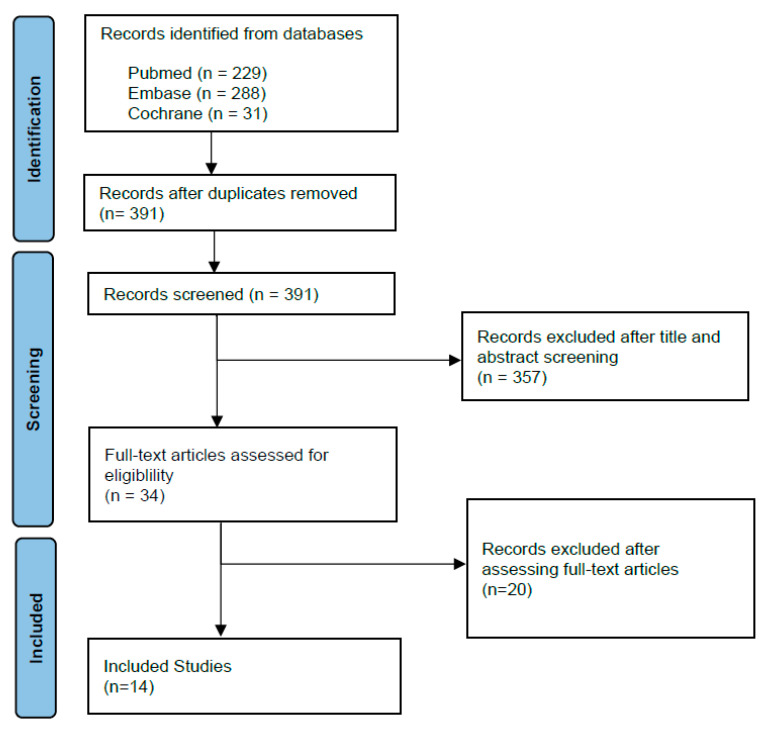
Flow diagram of included records.

We conducted a comprehensive review of the literature encompassing PARPi in prostate cancer. This systematic review was conducted according to the PRISMA (Preferred Reporting Items for Systematic Reviews and Meta-Analyses) guidelines. In November 2022, we conducted a search in the PubMed, Embase and Cochrane databases. MeSH terms and keywords “PARP inhibitors and prostate cancer” were used in searches in PubMed, Embase and Cochrane. In the initial search, we obtained 184, 278 and 30 articles in the PubMed, Embase and Cochrane databases, respectively. The initial queries were limited to papers published between 2012 and 2022. We then limited the search to papers that were written in English, or papers with available English translation. Duplicates were removed from the remaining selection. Two of the authors conducted screening of the remaining 391 publication abstracts. Case reports, editorials, reviews, letters, retrospective studies were excluded from the PubMed, Embase and Cochrane queries. Thirty-four articles were then chosen for full-text reading. We selected randomized controlled trials to attempt to limit potential biases and confounders. We excluded phase I randomized controlled trials. We selected phase II and III clinical trials in men with metastatic prostate cancer for their greater clinical impact and larger cohort sizes. In selected trials, patients in the intervention group were to receive a PARPi either alone or with other potentially complementary antineoplastic agents. Regular consensus meetings were held by the authors for discussion and information extraction from the selected manuscripts. Selected trials had efficacy and toxicity data. Efficacy data were reflected through outcomes such as objective response rates (ORR), radiologic progression-free survival (rPFS), disease-free survival (DFS) or overall survival (OS). Evaluation of side effects by a standard severity grading system such as the common terminology criteria for adverse events (CTCAE) was also required. All disagreements about selection and study inclusion between authors were discussed and resolved at consensus meetings with all authors (Figure 1).

## 3. Results

### 3.1. Biology

mCRPC is a complex disease state. Metastatic prostate cancer can originate from hormone-sensitive disease (mHSPC) whose growth is mediated through activation of the androgen receptor. Hence, mHSPC can be exquisitely sensitive to androgen deprivation therapy (ADT), though with disease progression, selective clonal pressures can stimulate gene mutations enabling castration resistance. However, mCRPC can also emerge from progression of nmCRPC preceded by nmHSPC disease. Castration resistance is classically defined as either clinical disease progression, three consecutive PSA rises in a castrate state (i.e., total testosterone < 50 ng/dL), or a PSA value above nadir + 2.0 ng/mL in a castrate state [17].

Several castration resistance mechanisms have been identified so far. AR amplification is one suggested method [18]. Despite androgen blockade, low levels of androgens may persist in circulation. Some CRPC clones may amplify the production of AR receptors [19,20], hence rendering them hypersensitive to minute background levels of androgens. Further studies supporting this mechanism have revealed changes in the genes encoding AR receptors making them more potent at detecting circulating androgens [21]. These changes, in turn, can lead to activation of a hypersensitivity pathway leading to disease progression [17]. Alternatively, the role of AR co-activators has been evaluated. AR co-activators and co-repressors have been determined to be upregulated or downregulated in CRPC, respectively, leading to increased androgen-induced gene transcription and disease progression [22,23]. Aberrant activation of the AR pathway through crosstalk is yet another observed mechanism. This progression pathway is a ligand-independent pathway through which growth factors and cytokines such as NF-KB [24], PI3K [25] or IGF [26] can promote increased AR signaling. Furthermore, CRPC state is often paradoxically associated with high levels of intra-tumoral androgens despite low levels of circulating androgens. Some theories suggest that, with suppression of the hypothalamic-pituitary-gonadal axis, CRPC relies on elevated androgen precursors such as dehydroepiandrostenedione (DHEA) produced by the adrenal glands. Even with androgen biosynthesis inhibitors, these adrenal androgens have proven to be resilient to suppression [27]. Thus, with DHEA transforming into 5a-androstenedione and dihydrotestosterone, CRPC can develop alternate pathways, bypassing testosterone altogether, and continue to unrepentantly promote tumor cell proliferation [28]. Conversely, castration resistance has been associated with aberrant activation of the KEGG metabolic pathway, which plays a role in glycolysis [29]. In addition, the contribution of splice-AR variants has been studied and is thought to play a role in post-translational control [17,30].

### 3.2. DNA Repair Genes

The induction of these DNA changes leading to castrate resistance is associated with an increased mutational burden. Subsequently, this mutational pressure can be associated with an increased risk of DNA damage. As such, appropriate repair genes become necessary for cancer cell survival. Consequently, there has been growing interest in targeted therapy for growth factor signaling and DNA repair pathways. Several studies using next-generation sequencing have allowed to better understand the genomic landscape of metastatic prostate cancer. Indeed, Pritchard et al. determined that many metastatic prostate cancer patients had DNA-repair gene mutations such as BRCA1, BRCA2, ATM, CHEK1/2, RAD51 [31] and that these mutations were present in 11.8% of prostate cancer patients. BRCA2 appeared to be the most common HRR mutation in prostate cancer with a prevalence of approximately 5% [31]. Dan et al. [32] discovered mutations in DNA damage repair genes among 22.7% of prostate cancer patients in his study, whereas Abida et al. [33] detevted similar mutations in 27% of their patient population. Mutations in HRR genes can be characterized as germline or somatic [34]. Nearly half of HRR mutations are thought to stem from a germline origin, whereas the rest are thought to be of somatic origin. Furthermore, while mutations in DNA repair genes occur in both localized and advanced prostate cancer, their occurrence is more frequent in metastatic vs. localized tumors (27% vs. 10%) as demonstrated by Armenia et al. [35] (Table 1).

### 3.3. DNA Repair Pathways

Homologous recombination repair (HRR) is a high-fidelity DNA repair pathway that repairs different types of DNA damage such as double-strand breaks (DSB), single strand DNA gaps and interstrand crosslinks. Repair of DSBs are particularly critical given that a single DSB can potentially lead to cell death [39]. Several genes such as BRCA1, BRCA2, PALB2 and RAD51D play important roles in this complex repair mechanism. HRR is initiated by the identification of DNA damage by ATM and ATR which can then phosphorylate proteins such as BRCA1, p53, H2AX and Chk2 [40]. Then, MRE11-RAD50-NBS1 (MRN) complexes arrive at sites of DNA damage and proceed to resect these areas. RAD50 structurally holds the damaged DNA ends and processes the 3′ end to create single-stranded DNA [41]. BRCA1 and BRCA2 are tumor suppressor genes that help prevent genomic instability. BRCA1 is bound and phosphorylated by ATM kinase after DNA damage. Thereafter, BRCA1, in association with BARD1 and BRIP1 proteins, help to constitute scaffolding necessary for additional repair proteins [40]. BRCA1 activates p53 binding protein 1 (53BP1) dephosphosphorylation through the phosphatase PP4C, which subsequently promotes the HRR pathway over the non-homologous end-joining (NHEJ) repair pathway [42]. BRCA2 acts as a recombination mediator by displacing RPA while loading and stabilizing polymerized RAD51 to sites of DSBs [43]. RAD51 proteins play an integral role for recombination during mitosis, meiosis and during HRR for DSBs [44]. RAD51 coat single-strand DNA to form a nucleoprotein filament that invades and pairs with a homologous region in duplex DNA. This juxtaposition leads to strand exchange.

Hence, HRR is dependent on BRCA1/2. Unrepaired SSB can develop into DSB during the formation of the DNA replication fork [45]. These errors can be repaired through BRCA-mediated HRR, but in their absence the replication fork cannot be restarted and will collapse over time, leading to genomic instability, cell cycle arrest and ultimately cell death [46].

Given these findings, homologous recombination repair (HRR) deficiency can be considered as an actionable pathway, given its essential role in double strand DNA break repair and its exploitability by PARPi. However, targeting based on deficiencies in different DNA repair genes can produce different results, given that their role in HRR is not equal, and given some genes such as FANCA, CHEK2 and ATM are involved in different pathways. FANCA plays a role in DSB repair single-strand annealing sub-pathway by catalyzing single-strand annealing and strange exchange [47]. MRE11/RAD50/NBS1 can sense DSBs and recruit ATM to the damaged sites. ATM further promotes H2AX phosphorylation near DSBs [48]. CHEK2 encodes a serine–theronine kinase which oversees a DNA damage checkpoint pathway. Chk2 is activated by ATM in response to double strand breaks. Phosphorylated Chk2 kinase can act as a signal transducer and can itself phosphorylate other proteins such as Cdc25 phosphatases, p53, PML, E2F-1 and BRCA. Hence, CHEK2 and ATM play important roles in DNA repair and apoptosis [49].

### 3.4. PARP

PARP is a family of enzymes which can catalyze the transfer of ADP-ribose on target proteins. PARP proteins affect different cellular functions including chromatin modulation, transcription, replication and DNA repair [50]. Indeed, the variety of proteins and involvement in different pathways of this family of proteins can explain some off-target effects of PARP inhibition. PARP proteins are composed of a DNA binding domain (DBD), an automodification domain (AD) and a catalytic domain (CD). Notably, PARP1 and PARP2 play an important role in single strand break (SSB) repair and are a target of choice for novel therapies.

PARP1 identifies and binds to sites of single strand DNA damage. SSBs are the most common form of DNA damage found in cells. They are induced by the breaking of the phosphodiester bond between DNA forming deoxyriboses. While generally more numerous, these lesions do not, by themselves, cause cell death [51]. When PARP1 attaches to these DNA lesions through its DBD, PARP1 activates and synthesizes poly-ADP-ribose (PAR) by using nicotinamide adenine dinucleotide (NAD+) as a substrate and forming nicotinamide as a by-product.

PAR is then transferred to acceptor proteins including PARP itself at its AD, a process known as autoPARylation [52]. PARP1 also helps generate PAR chains that bind to other acceptor proteins such as histones. This process is known as PARylation and helps generate a DNA damage response through enhanced signaling and repair factor recruitment. Thus, PARP1, through post-translational modifications, at the site of DNA lesions, can form a docking platform for other DNA repair factors [53]. Amongst others, AutoPARylated PARP1 can recruit histone remodeling enzymes, mobilize the DNA scaffolding protein XRCC1, and can recruit, through PAR, the nucleosome repositioning enzyme ALC1, hence forming part of the DNA SSB repair machinery. Subsequently, DNA polymerase beta and ligase III will be mustered to the areas of DNA damage [54,55]. However, when extensive chains of PAR are formed, it confers a large negative charge to PARP1, which promotes DNA detachment through repulsion, thus deactivating PARylation [56]. Consequently, after the release of PARP1, other proteins can access and pursue the DNA repair process [57]. PAR is subsequently rapidly degraded by other enzymes such as PAR glycohydrase or PAR hydrolase. In the absence of these enzymes, the accumulation of PAR can be cytotoxic. While PARP plays a major role in SSB repair by forming a base excision repair (BER) complex, it plays an additional role in nucleotide excision repair and mismatch repair through the PARylation of XPA and MSH6, respectively [58]. PARP also plays a role in double strand breaks (DSB). Although DSBs occur less frequently, they are harder to repair and can lead to genomic instability and cell death. Repair of DSB is overseen by non-homologous end-joining (NHEJ) and the high-fidelity homologous recombination repair processes (HRR). PARP proteins intervene in both these repair process by PARylating DNA-PKcs [59], recruiting MRE11 [60] and activating ATM [61], a major DSB signaling kinase. Similarly to its activity in SSBs, PARP1 senses DNA DSBs and produces PAR proteins near damage sites promoting chromatin relaxation, histone displacement and enhancing repair factor recruitment [62]. Thus, PARP-1 grants access to the resection machinery. Repair pathway choice can be determined by DNA end resection which is initiated by MRN-CtIP. The latter creates a 3′ single-stranded DNA which permits recruitment of homologous repair proteins that conversely prevent the initiation of NHEJ repair pathway [63,64].

### 3.5. PARP Inhibitors

A better understanding of PARP activity has led to therapies seeking its inhibition, given that DNA repair and replication are essential for malignant cell survival and proliferation. SSBs are the most common form of DNA damage and are usually repaired through base excision repair, nucleotide excision repair or mismatch repair systems. Hence, PARPi, by impeding the role of PARP enzymes, significantly impair SSB repair. Thus, it was theorized that the accumulation of damage through SSBs would ultimately lead to cell death through synthetic lethality. This phenomenon occurs when an error in either one of two genes has little effect on an organism, whereas a combination of defects in both genes results in its death. However, evidence that synthetic lethality can be attributed to accumulation of SSBs is limited [65].

**Figure 2:**
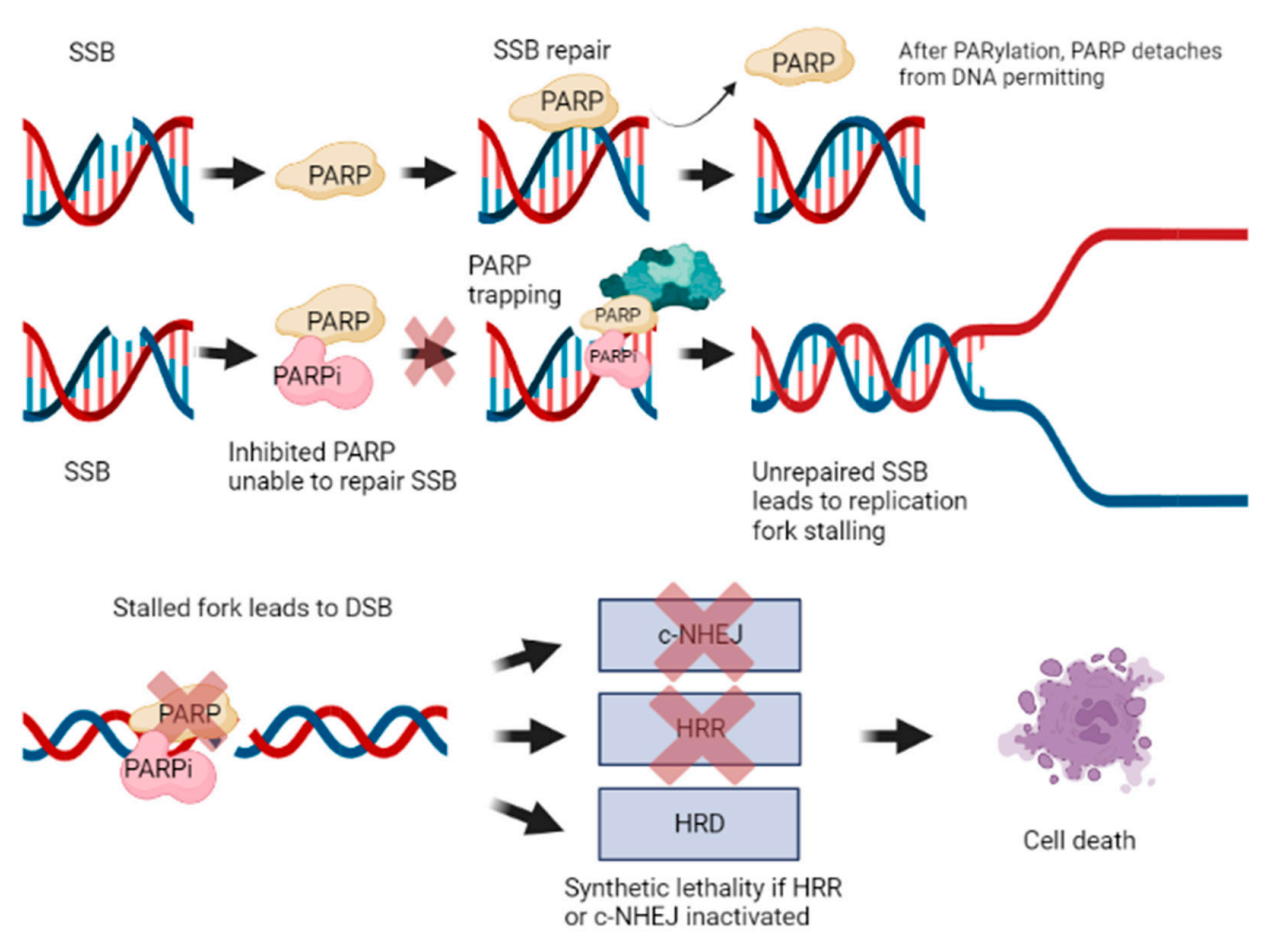
Mechanism of action of PARPi and synthetic lethality. This image was created using biorender.com.

Rather, it was theorized that synthetic lethality may be due to stalled replication forks converted to DSB. Indeed, SSBs that remain unrepaired become DSB when the replication fork moves through; however, these DSBs are one-ended, hence they cannot be repaired by NHEJ, making the cells dependent on homologous repair. Accumulation and inability to repair these DNA DSBs in the context of absent or impaired HRR apparatus can culminate in cell death [66,67]. In fact, synthetic lethality is a phenomenon that occurs when an error in either one of two genes has little effect on an organism, whereas a combination of defects in both genes results in its death (Figure 2).

PARP1 is the major target of PARPi. However, given its structural similarities, PARP2, PARP3 and other off-target interactions with other PARP enzymes can also occur [65,68,69]. Studies in PARP regulation have revealed that the by-product Nicotinamide exerts a mild inhibitory effect by acting directly at the catalytic domain of PARP1 [70,71,72,73]. While it is one amongst many regulators of PARP, it has served as a model for developing high affinity synthetic inhibitors, which compete with NAD+. PARPi have several mechanisms of action [65]. PARPi retain PARP1 at DNA lesions by competing with NAD+ at the catalytic binding site [56]. PARPi can trap PARP enzymes on SSB leading to subsequent replication stress [74]. As described previously, autoPARylation causes PARP1 to dissociate from DNA. However, given its affinity at sensing and interacting with damaged DNA, PARP1 can remain bound to DNA in the presence of a PARP inhibitor due to its inability to perform the PARylation reaction, and consequently dissociate [75]. Alternatively, another hypothesis for PARP trapping stems from the reverse allosteric theory. This mechanism proposes that PARPi binds to NAD+ and thus develops enhanced affinity to DNA through PARP’s zinc finger domain. This is exemplified through allosteric modulation of the ZN1-WGR-HD and WGR-Zn3-HD interfaces of PARP1 [68]. Trapped PARP on damaged DNA prevents DNA repair, stabilizes PARP-based toxic repair complexes and leads to degeneration of stalled replication forks on DNA DSBs [56,76].

The rationale for research into PARPs is multifactorial. Cancer cell death has been linked to DSB. In the absence of functional HRR, more SSB can be converted into DSB leading to biological cell death. PARPi has the potential to enhance standard therapies through a more personalized targeted approach. PARPi are also naturally a therapy of choice against cancers exhibiting DNA repair deficiencies such as homologous recombination deficiency. Similarly, PARPi can be beneficial for patients with hereditary syndromes impairing DNA repair such as BRCA1 or BRCA2 [77]. Furthermore, whereas HRR gene mutations can induce prostate cancer by increased genomic instability, prostate cancers with these mutations have also been determined to be more aggressive, rapidly progressive and have a greater metastatic potential [78]. Hence, PARPi may be of particular benefit to this subgroup of prostate cancer patients. While it is clear that synthetic lethality drives the benefit of PARPI, studies have also started exploring whether synergistic combinations with other antitumoral agents can overwhelm existing DDR mechanisms and further enhance cancer cell death.

### 3.6. Molecular Testing

Integration of molecular testing has led to improved cancer outcomes in oncology. In prostate cancer, molecular testing aims to both optimize and personalize patient care. HRR gene testing can be beneficial in prostate cancer patients. It can help identify underlying familial risk of cancers, serve as a prognostic marker for aggressive disease and act as a predictive marker for PARPi. In fact, several reputable sources recommend integration of genomic testing in prostate cancer such as the NCCN [79], AUA/ASTRO/SUO [80], ESMO [81,82] and EAU [83] guidelines. Currently, the HRR mutations can be identified through blood, tissue and plasma samples. However, tissue testing remains the gold standard. Furthermore, studies have demonstrated the HRR mutations are often early stable events, which endorses archival tissue sampling and consequently may minimize the need for new painful patient biopsies [84].

### 3.7. Studies

Currently, most research on PARPi in prostate cancer revolves around four agents: olaparib, niraparib, rucaparib and talazoparib. So far, the FDA has approved olaparib and rucaparib for the treatment of mCRPC based, respectively, on the results of the PROFOUND and TRITON studies in 2020 [85]. Rucaparib is approved for *BRCA1*/2 mutations after progressing on an ARAT (androgen receptor axis-targeted therapy) and taxane-based chemotherapy, whereas olaparib is indicated for patients with any HRR gene mutations post-ARATs. Niraparib has received the FDA’s breakthrough therapy designation based on promising results seen in the GALAHAD trial. Meanwhile, talazoparib combined with enzalutamide has recently been assigned a priority review by the FDA based on findings of the TALAPRO-2 study [86]. Although PARPi act similarly on a biologic level, they are known to have different trapping efficiencies. Talazoparib has a trapping efficiency nearly one hundred times more potent than that of niraparib, followed by olaparib and rucaparib [87,88,89]. While higher trapping efficiency may potentially be more effective, it has been associated with more toxicity [90].

#### 3.7.1. Olaparib

The TOPARP-A study [37] was an adaptive phase II trial evaluating olaparib in the mCRPC setting. Patients received olaparib until disease progression, intolerable toxicity or withdrawal from the study. TOPARP-A sought primarily to assess the response rate, which was defined radiologically per RECIST criteria or biochemically by a PSA decreased by ≥50%. Enrolled patients had all been previously treated with docetaxel, most (98%) had received an ARAT (i.e., abiraterone or enzalutamide) and roughly 60% had received cabazitaxel prior. DNA sequencing was performed to identify DNA repair gene mutations such as BRCA, ATM, CHEK2 and FANCA. These mutations were identified in one third of patients. Median OS was 13.8 vs. 7.5 months. Interestingly, while the overall response rate was 33%, the response rate amongst patients with mutations was 88%, and 6% in the remainder.

Subsequently, the TOPARP-B [91] phase II trial was initiated. This study assessed olaparib in mCRPC patients pre-selected for DDR gene defects. Patients received either 300 or 400 mg in a “pick the winner” design. The primary endpoint was either radiological or PSA response. Overall, the response rate was 54% in the 400 mg cohort and 37% in the 300 mg cohort. Progression-free survival (PFS) was 5.4 months. Response rates per DDR gene defects were, respectively, 80% for *BRCA1*/2, 57% for *PALB2*, 37% for *ATM*, 25% for CDK12, and 20% for other identified genes.

Hence, the TOPARP trials helped to identify the anti-tumor activity of olaparib in mCRPC, the importance of DDR gene defects for therapy, the differential response per variable DDR gene defects and the importance of dose.

Building on the foundations of the TOPARP trials, the PROFOUND [92] study sought to crystallize their results in a phase III randomized controlled trial (RCT). PROFOUND randomized pre-selected patients with DNA repair gene defects to either olaparib or second-line ADT in the form of an ARAT (abiraterone/enzalutamide). Patients in the experimental arm received olaparib 300 mg PO BID. Prior taxane therapy was permitted. Patients were stratified into two groups. Cohort A included patients with *BRCA1*, *BRCA2* and *ATM* mutations. Cohort B included patients with 12 other DDR gene mutations. Then, in each group, patients were randomized to receive olaparib or an ARAT. The primary endpoint for this trial was radiological PFS (rPFS) based on RECIST 1.1 or PCWG3 criteria. DRR gene defects were identified through tissue testing, while circulating tumor DNA (ctDNA) and germline samples were retrieved for a planned retrospective analysis. PROFOUND ultimately enrolled 387 eligible patients. In Cohort A, olaparib significantly reduced rPFS by two thirds (median PFS 7.39 vs. 3.55 months; HR 0.34). Furthermore, a significant rPFS benefit was also seen in in Cohorts A + B (5.82 vs. 3.52 months; HR = 0.49; *p* < 0.001). In Cohort A, patients also had a significant benefit in time to pain progression and overall survival (19.09 vs. 14.69; HR0.69; *p* = 0.02). A gene-by-gene analysis additionally identified that patients with BRCA gene mutations benefitted most from olaparib. Amongst patients on olaparib, over 20% of patients experienced ≥ grade 3 anemia, while rates of ≥grade 3 neutropenia or thrombocytopenia were <5%. One case of acute myeloid leukemia was identified and pneumonitis was reported in four patients, of which half were in the control group.

The PROPEL [93] study sought to identify whether there was a benefit when combining abiraterone and olaparib as first-line treatment for all-comers in the mCRPC setting. While ARATs alone were a standard of care of first-line mCRPC, it has been theorized that a synergy can exist between both agents, given that ARATs can induce HRR deficiencies and PARPi can increase the activity of ARATs through AR-dependant transcription [93]. While patients who had received prior ARATs were excluded, patients who had received docetaxel were not. Patients received 1000 mg of abiraterone daily and olaparib 300 mg PO BID. Patients were randomized between olaparib and abiraterone vs. placebo and abiraterone. The study reported a median rPFS of 24.8 vs. 16.6 months favoring the experimental arm, thus conferring a 34% reduction in progression or death. Data for overall survival (OS) had not yet reached maturity, but seemed to show a trend favoring the experimental arm (HR 0.86; CI 0.66–1.12; *p* = 0.29). Presence of HRR mutations was at approximately 30% in each arm. The safety profile of this drug combination appeared consistent with individual agents. The most common adverse effect in the experimental arm was anemia in 46%. While grade ≥ 3 adverse events (AE) (47.2 vs. 38.2%) and discontinuation of treatment (13.8 vs. 7.8%) were more frequent in the experimental arm, death due to AE was similar between arms (4.0 vs. 4.3%) (Table 2).

#### 3.7.2. Niraparib

Niraparib, a selective PARPi that inhibits PARP1 and PARP-2, was also tested in the mCRPC setting. The Galahad study [97], a phase II, single-arm trial, aimed to primarily describe an objective response to niraparib based on RECIST 1.1 and PCWG3 imaging findings among patients having progressed on ARATs and Taxotere with BRCA mutations. The trial enrolled 289 patients and obtained a response rate of 34.2% in patients with *BRCA1*/2 mutations.

Subsequently, the Magnitude [98] phase III RCT compared the combination of niraparib and abiraterone to abiraterone alone. While participants were tested for HRR gene mutations, patients could be treated irrespective of their status. Patients were treatment-naïve in the mCRPC setting, but were stratified for prior use of taxanes in the mHSPC setting. Patients received niraparib 200 mg PO OD and abiraterone 1000 mg PO OD. In total, 1000 patients were recruited, of which 40% had a HRR mutation. The primary endpoint was rPFS. A prespecified early futility analysis, based on a composite index of RPFS or PSA progression, demonstrated no benefit for the experimental arm in patients without HRR mutations. In the HRR mutation positive experimental arm, rPFS (16.5 vs. 13.7 months) was significantly improved at a median FU of 16.7 months. This corresponded to a reduction in the risk of progression or death of 27%. OS data were also immature, but suggested a trend to improved HR of 0.94 (95% CI 0.65–1.36; p = 0.73). The overall response rate favored the experimental arm in patients with HRR mutations (60 vs. 28%). Adverse events were more frequent in the experimental arm (Table 3).

#### 3.7.3. Rucaparib

Rucaparib was examined in similarly designed trials for mCRPC. The Triton-2 [18], single arm, open-label phase II study assessed the use of rucaparib in mCRPC patients with a BRCA gene defect who had previously been exposed to ARATs and docetaxel. Patients received rucaparib 600 mg PO BID. The study enrolled 115 patients and obtained an overall response rate of 43.6 to 50.8% and a median rPFS of 9.0 months.

The Triton-3 [99] study compared mCRPC chemotherapy-naïve patients with BRCA mutations receiving physician’s choice standard of care treatments (enzalutamide/abiraterone/docetaxel) vs. rucaparib 600 mg PO BID in mCRPC patients. The trial enrolled 405 patients. Median PFS favored the experimental arm (10.2 vs. 6.4 months; *p* = 0.0003), which implied that, in this setting, rucaparib may be better than chemotherapy. The safety profile of rucaparib was similar to other PARPs. The most common AE ≥ grade 3 was anemia. (Table 4).

#### 3.7.4. Talazoparib

The Talapro-1 [101] study was a phase II study assessing the efficacy and safety of talazoparib in mCRPC progressing after chemotherapy and ARATs. Eligible patients were screened to have one of 11 possible HRR gene deficiencies. Participants received talazoparib 1 mg (or 0.75 mg for kidney dysfunction). The study recruited 148 patients. The objective response rate per RECIST 1.1 was 28% in all comers and 43.9% of BRCA1/2 mutations were present.

The Talapro-2 [102] study is a phase III study comparing enzalutamide combined with either talazoparib or placebo in new mCRPC. The study enrolled 1095 patients, 750 were recruited to the all-comer cohort, while 380 with DDR gene defects were analysed in a separate cohort. Patients received enzalutamide and either talazoparib or a placebo. The primary endpoint was rPFS. While results have not been officially posted, it has been reported that rPFS exceeded the pre-specified HR of 0.696 and that there was a trend to an OS benefit (Table 5).

#### 3.7.5. Immunotherapy Combinations

PARPi have also been assessed in combination with immunotherapy. The rationale for this combination stems from a theorized immune activation through enhancement of stimulator of interferon genes (STING). A phase II trial [94] enrolled 17 patients with mCRPC for treatment with Durvalumab, an immune checkpoint inhibitor and olaparib. PSA or radiologic progression occurred in 53% of patients [95]. CHeckMate 9KD [100] is a phase II trial assessing the safety and efficacy of nivolumab, a PD-1 inhibitor, in combination with rucaparib, docetaxel or enzalutamide in patients with mCRPC. Patients in cohort A received nivolumab 280 mg administered intravenously (IV) every 4 weeks with rucaparib 600 mg PO BID. Patients were stratified by prior exposure to docetaxel. The study determined the combination to be active in patients with homologous recombination deficiency (HRD) and BRCA 1–2 mutations. The main adverse effects were nausea and anemia. The multi-arm Keynote-365 study [103] aimed to assess the safety of pembrolizumab, a PD-L1 inhibitor, in mCRPC through a phase IB-II trial. The drug was combined with other antitumoral agents in nine cohorts (Arms A-I). Cohort A compared the efficacy of combining pembrolizumab with olaparib. Recruited patients received pembrolizumab 200 mg IV on day 1 of Q3 week cycles with olaparib 300 or 400 mg PO BID given continuously. In total, 102 enrolled patients were treated on this arm. The study determined primarily that the PSA response rate was 15%, the objective response rate per RECIST1.1 was 8.5% in patients with measurable disease (58%). rPFS was 4.5 months and median OS was 14 months. Grade 3–5 adverse advent occurred in 49 patients including six treatment-related deaths. The KEYLYNK-010 [96] phase III RCT evaluated the combination of pembrolizumab and olaparib vs. either enzalutamide or abiraterone in mCRPC. However, at interim analysis, the study was stopped due to futility, given rPFS was not improved and the combination seemed to be associated with higher grade 3–5 toxicity in the experimental arm.

## 4. Discussion

PARPi have become an important focus of research in the mCRPC setting. Over the last decade, it has become evident that mCRPC is associated with an increased occurrence of mutations in DNA repair genes. The existence of these deficiencies creates a window of opportunity to target mCRPC through novel agents. The critical role PARP proteins play in maintaining the remaining critical alternate DNA repair pathways has made it a target of interest. Our improved understanding of DNA repair pathways has permitted us to exploit this weakness in cancer cells with DDR gene mutations to induce cancer cell death. PARPi thus attests to an evolution in treatment readily integrating a more personalized approach.

With many trials nearing their term, it is clear that PARPi are a promising therapeutic avenue. Many phase II clinical trials have found favorable overall responses in patients with HRR mutations and have demonstrated that these agents have significant activity in mCRPC. They have also demonstrated that these agents, for the most part, have a safe toxicity profile, with the most serious adverse effects being associated with expected hematological toxicity [91,101,104].

Subsequent phase III trials have demonstrated that PARPi, across the board, can improve response rates and rPFS in patients with mCRPC [93]. These trials have demonstrated that the combination of ARATs with PARPi is safe and effective, and therefore should be considered a new standard of care in mCRPC. While the data presented so far are promising, current trials have not attained maturity and are unable confirm an overall survival benefit [98,102,105].

However, in the data presented so far, there remains ongoing controversies. An unresolved question revolves around the necessity of HRR mutations for PARPi to be effective in mCRPC. All the phase II and phase III trials have shown that PARPi generates good responses when HRR mutations are present, more so if there are defects in *BRCA1*/2. However, whereas PROPEL [93] indicated that this benefit could be extrapolated to all-comers, MAGNITUDE [98] did not, given its all-comer arm stopped early after a futility analysis. While both studies had similar objectives, it is important to note that they had different designs. While the PROPEL and MAGNITUDE trials targeted disease in the first-line mCRPC setting, PROPEL excluded patients who had previously had chemotherapy or ARATs, whereas MAGNITUDE included them if they were treated with those agents in the mHSPC state. Hence, it is possible that the results correspond to two ultimately different patient populations, or that there may be a lead-time bias in some cases. Another explanation for this occurrence was that the futility analysis may have been flawed, given 83/148 events in the composite endpoint were due to PSA failure [106]. Alternatively, it is possible that there may exist differences in drug effectiveness, or at least in optimal drug doses. While higher doses can be associated with improved responses, they can also lead to greater toxicity [107].

While the use of PARPi has been thoroughly explored in the mCRPC setting, trials are now seeking to assess its benefits in other disease phases. Indeed, DDR mutations can be present in earlier disease stages, and thus remain a target of opportunity. Furthermore, comparably, the efficacy of ARATs was initially evaluated in the mCRPC setting, before becoming standard of care in the mHSPC setting, and showing promise in the high-risk/locoregional setting. Improvements in the mHSPC setting have significantly improved the prognosis of metastatic prostate cancer patients. The Talapro-3 trial is currently assessing the role of enzalutamide with talazoparib in patients with DDR gene mutations in mHSPC. Talapro-3 trial [108] aims to recruit 550 patients with alterations in 12 possible DDR genes. This study will also primarily look at rPFS, as well as OS. On the other hand, AMPLITUDE [109] assesses the role of niraparib in combination with abiraterone acetate and prednisone for the treatment of patients afflicted with mHSPC with deleterious germline or somatic alterations of homologous recombination repair genes. Like Talapro-3, this study will primarily examine rPFS and OS as other efficacy assessment.

The combination of PARPi with other antitumoral agents can be beneficial. There has been interest in combining PARPi with radionuclide therapy. Both agents are used in the mCRPC setting and have potential synergy [110]. The rationale for their combination lies in the potential for increasing and sustaining DNA DSBs. This radiosensitization relies on blocking repair through PARP and overwhelming any remaining DNA repair pathways [109]. While the combination is promising, patients will require close monitoring given the hematological and gastrointestinal side effects of PARPi. Furthermore, radionuclides therapies have been associated with nephrotoxicity, while PSMA targeting agents can induce salivary gland damage. The COMRADE trial [110] is an ongoing phase I/II trial assessing the benefits of olaparib with Radium-223 in mCRPC with bone metastasis. For the phase I portion, 12 patients were enrolled. As per the dose escalation protocol, the trial so far has determined that it is safe to combine Radium-223 with olaparib 200 mg PO BID. In the same manner, the ongoing Nirarad trial is a phase IB study assessing the tolerance dose of niraparib with Radium-223 [111].

Conversely, trials have sought to assess the role of PARPi with radiotherapy [97,98,99]. In a similar manner to radionuclide therapy, PARPi is hypothesized to enhance cellular radiosensitivity [112,113]. Radiotherapy, while aiming to induce DSB and cancer cell death, mostly creates SSB, which can be easily repaired by SSB DNA repair genes [114]. However, with PARPi, BER pathways can be compromised leading to collapsed replication forks [115], which can ultimately lead to an enhanced conversion of SSB to DSB [116]. Thus, inhibition of PARP enzymes in an environment of substantial SSB can overwhelm remaining DNA repair mechanisms in cancer cells [117]. Furthermore, radiation-induced DNA double-strand lesions in prostate cancer cells activate the AR axis [118]. This, in turn, can lead to the upregulation of several DDR genes. ADT may induce the downregulation of these DDR genes, promoting increased cancer cell death [119]. Consequently, subsequent increased PARP activity can lead to tumor-cell survival and modulation of AR-axis activity [90], which in turn can be countered by PARP inhibition.

The recently approved Canadian PR23 phase II trial will seek to evaluate the impact of this combination treatment in the mHSPC setting. Indeed, PR23 will randomize patients to SBRT with or without niraparib in mHSPC patients treated with ADT and ARATs.

The ASCLEPlus [120] phase I/II trial is currently recruiting patients with localized prostate cancer. It is assessing the role of SBRT with abiraterone and leuprolide and aims primarily to assess the max tolerable dose of niraparib with SBRT and evaluate bPFS with this regimen. MD Anderson is also conducting a trial assessing ADT with or without niraparib after radiotherapy for high risk localized or locally advanced prostate cancer (NCT04947254). The NADIR NRG phase II trial is also recruiting patients to evaluate the benefit of adding niraparib to standard external beam radiotherapy with long-term androgen deprivation for high-risk patients (NCT04037254) [121].

## 5. Conclusions

PARPi is an effective novel targeted therapy in prostate cancer. Several trials so far have demonstrated encouraging results when used in the mCRPC setting. While its use as monotherapy has shown good response rates in late-stage disease, its combination with other anti-tumoral agents is currently being investigated and remains promising. The ongoing trials in mCRPC have aimed to confirm survival benefits and several new trials are currently seeking to expand the use of this agent in earlier disease phases by further exploiting synthetic lethality and overwhelming cancer cell DNA repair mechanisms.

## Figures and Tables

**Table 1:** Mutations in DDR Genes in prostate cancer.

Gene Mutations	Prevalence in Localized Prostate Cancer (%) [36]	Incidence of Germline Mutations in Metastatic Prostate Cancer (%) [31,37]
Overall	8–10	11–33 [38]
*ATM*	2.7	1.6–7.3 [31,37]
*BRCA1*	0.7	0.9 [31]
*BRCA2*	2.0	5.3–13 [31,37]
*CDK12*	1.5	5 [37]
*FANCA*	0.6	0.3 [37]
*CHEK*2	0.1	1.9 [31]
*RAD51*	0.1	0.3 [37]

**Table 2:** Trials evaluating the role of olaparib in prostate cancer.

TRIALS	Type	Patient Selection and Arms	Patients (N)	Median FU (Mo)	Outcomes	Toxicity
TOPARP-A [37]	Phase II	Unselected mCRPC 1: olaparib	50	14.4	Composite response rate (PSA and rPFS): overall:33% DDR gene mutations: 88%	107 serious AE in 49 patients. Most common: anemia
TOPARP-B [91]	Phase II RCT	Preselected mCRPC 1: olaparib 400 mg BID 2: olaparib 300 mg BID	92	17.6	PFS: 5.4 Months Response rate per DDR gene mutation: 20–80%	Most common G3–4 toxicity: anemia (31–37%)
PROFOUND [92]	Phase III RCT	Preselected mCRPC stratified per mutation (Cohort A: BRCA1, BRCA2, ATM vs. Cohort B: 12 other DRR gene mutations) 1: olaparib 2: abiraterone/enzalutamide	387	21.9	Median OS: 14.1 vs. 11.5 Mo. Median rPFS: Overall: 5.82 vs. 3.52 months; HR = 0.49 Cohort A: 7.39 vs. 3.55 months, favoring olaparib	≥G3 anemia: 21.5% ≥G3 neutropenia: 3.9% ≥G3 thrombocytopenia: 3.5%
PROPEL [93]	Phase III RCT	Unselected mCRPC 1: olaparib + abiraterone 2: placebo + abiraterone	796	NR	Median rPFS of 24.8 vs. 16.6	≥G3 AE: 47.2 vs. 38.2% Treatment D/C: (13.8 vs. 7.8%)
Karzai et al. [94]	Phase II	Unselected mCRPC 1: durvalumab + olaparib	17	9.7	Median rPFS: 16.1 Mo Overall composite response: 53%	G3 anemia in 23.5% G3 nausea in 11.8% G4 lymphopenia: 5.9%
Keynote-365 [95]	Phase I-II	Unselected mCRPC–multiple arms (A to I) ARM A: pembrolizumab + olaparib	102	24	ORR: median rPFS: 4.5 months median OS: 14 months	Grade 3–5 TRAE: 35% including anemia and nausea. TR-Death: n = 2
KEYLYNK-010 [96]	Phase III RCT	Unselected mCRPC 1: pembrolizumab + olaparib 2: enzalutamide or abiraterone	793	N/A	Stopped due to futility at interim analysis	

Preselected mCRPC: Patients preselected for DDR or HRR gene deficiencies, NR: Not reported, N/A Not associated, AE: Adverse events, TRAE: Treatment-related adverse events, ORR: Objective response rate, Composite response rate: based on PSA and radiologic progression, rPFS: radiologic progression-free survival, HHR: homologous recombination repair, DDR: DNA damage repair, mCRPC: metastatic castrate-resistant prostate cancer, OS: overall survival, G: grade, PE: Pulmonary embolism.

**Table 3:** Trials evaluating the role of niraparib in prostate cancer.

TRIALS	Type	Patient Selection and Arms	Patients (N)	Median FU (Mo)	Outcomes	Toxicity
Galahad [97]	Phase II	Preselected BRCA+ mCRPC 1: niraparib	289	10.0	Radiologic response rate: 34.2%	Most common AE: nausea (58%), anemia (54%), vomiting (38%) ≥G3 AE: 75%
Magnitude [98]	Phase III RCT	Unselected mCRPC, but stratified for HRR gene mutation. However, HRR- patient arm D/C after futility analysis 1: niraparib + abiraterone 2: placebo + abiraterone	423	16.7	rPFS 16.5 vs. 13.7 Mo Overall response based on composite index: 60 vs. 28%	≥G3 AE: 67.0% versus 46.4%. Drug-related AE: 76.4 vs. 55.0%. Most common AEs leading to dose reduction were anemia (13.2%) and thrombocytopenia (2.8%)

Preselected mCRPC: Patients preselected for DDR or HRR gene deficiencies, NR: Not reported, N/A Not associated, AE: Adverse events, TRAE: Treatment-related adverse events, ORR: Objective response rate, Composite response rate: based on PSA and radiologic progression, rPFS: radiologic progression-free survival, HHR: homologous recombination repair, DDR: DNA damage repair, mCRPC: metastatic castrate-resistant prostate cancer, OS: overall survival, G: grade, PE: Pulmonary embolism.

**Table 4:** Trials evaluating the role of rucaparib in prostate cancer.

TRIALS	Type	Patient Selection and Arms	Patients (N)	Median FU (Mo)	Outcomes	Toxicity
Triton-2 [18]	Phase II	Preselected BRCA+ mCRPC 1: rucaparib	115	17.3	Overall response: 50.8 vs 43.6% Median rPFS 9.0 Mo	≥G3 AE: Overall: Present in 60.9% fatigue (8.7%), anemia (25.2%), thrombocytopenia (9.6%), ALT/AST↑ (5.2%)
Triton-3 [99]	Phase III RCT	Preselected BRCA+ mCRPC 1: enzalutamide/abiraterone/chemo 2: rucaparib	405	17.1	Median rPFS: 10.2 vs 6.4 Mo	≥G3 AE: 76.4% vs 55.0% anemia: 23.7%, neutropenia: 7.4% fatigue: 7.0% thrombocytopenia: 5.9%
CHeckMate 9KD [100]	Phase II	Unselected mCRPC 1: nivolumab + rucaparib (Cohort A) Cohort A1/A2: prior chemo vs chemo naïve 2: nivolumab + docetaxel + prednisone 3: nivolumab + enzalutamide	A1: 88 A2: 71	A1: 11.9 A2: 17.5	A1: ORR: 10.3% ORR HRD+: 17.2% Median rPFS: 11.9 Mo A2: ORR: 15.4% ORR HRD+: 25.0% Median rPFS: 8.1Mo	A1: ≥G3 TRAE: 54.3% anemia: 20.5%, neutropenia: 10.2% A2: Grade 3/4 TRAE: anemia: 14.1%, 12.7%↑LFTs (7.0-12.7%),

Preselected mCRPC: Patients preselected for DDR or HRR gene deficiencies, NR: Not reported, N/A Not associated, AE: Adverse events, TRAE: Treatment-related adverse events, ORR: Objective response rate, Composite response rate: based on PSA and radiologic progression, rPFS: radiologic progression-free survival, HHR: homologous recombination repair, DDR: DNA damage repair, mCRPC: metastatic castrate-resistant prostate cancer, OS: overall survival, G: grade, PE: Pulmonary embolism.

**Table 5:** Trials evaluating the role of talazoparib in prostate cancer.

TRIALS	Type	Patient Selection and Arms	Patients (N)	Median FU (Mo)	Outcomes	Toxicity
Talapro-1 [101]	Phase II	Preselected mCRPC 1: talazoparib		16.4	Overall response: 28% Response in BRCA+: 43.9%	Serious TRAE: 34%, most common PE (6%), anemia (4%) Most common G3–4 TRAE: anemia (31%), thrombocytopenia (9%), and neutropenia (8%)
Talapro-2 [86]	Phase III RCT	Preselected and unselected cohorts mCRPC 1: enzalutamide + talazoparib 2: enzalutamide + placebo	1095	NR	rPFS exceeded the prespecified HR of 0.696	NR

Preselected mCRPC: Patients preselected for DDR or HRR gene deficiencies, NR: Not reported, N/A Not associated, AE: Adverse events, TRAE: Treatment-related adverse events, ORR: Objective response rate, Composite response rate: based on PSA and radiologic progression, rPFS: radiologic progression-free survival, HHR: homologous recombination repair, DDR: DNA damage repair, mCRPC: metastatic castrate-resistant prostate cancer, OS: overall survival, G: grade, PE: Pulmonary embolism.
